# Supplementary material for: Control of a hippocampal recurrent excitatory circuit by cannabinoid receptor-interacting protein Gap43
Source: Nat Commun. 2023 Apr 21;14:2303. doi: 10.1038/s41467-023-38026-2 (PMC10121561; doi:10.1038/s41467-023-38026-2)
Supplement: Supplementary file 1 — Supplementary Information [file 41467_2023_38026_MOESM1_ESM.pdf]

SUPPLEMENTARY INFORMATION

CONTROL OF A HIPPOCAMPAL RECURRENT EXCITATORY CIRCUIT BY CANNABINOID RECEPTOR-INTERACTING PROTEIN GAP43

Irene B. Maroto[1,2,3], Carlos Costas-Insua[1,2,3], Coralie Berthoux[4], Estefanía Moreno[5], Andrea Ruiz-Calvo[1,2,3], Carlos Montero-Fernández[1], Andrea Macías-Camero[1], Ricardo Martín[1,6], Nuria García-Font[1,6], José Sánchez-Prieto[1,6], Giovanni Marsicano[7], Luigi Bellocchio[7], Enric I. Canela[5], Vicent Casadó[5], Ismael Galve-Roperh[1,2,3], Ángel Núñez[8], David Fernández de Sevilla[8], Ignacio Rodríguez-Crespo[1,2,3], Pablo E. Castillo[4,9], Manuel Guzmán[1,2,3,*]

[1]Department of Biochemistry and Molecular Biology, Instituto Universitario de Investigación Neuroquímica (IUIN), Complutense University, Madrid 28040, Spain
[2]Centro de Investigación Biomédica en Red sobre Enfermedades Neurodegenerativas (CIBERNED), Instituto de Salud Carlos III, Madrid 28029, Spain
[3]Instituto Ramón y Cajal de Investigación Sanitaria (IRYCIS), Madrid 28034, Spain
[4]Dominick P. Purpura Department of Neuroscience, Albert Einstein College of Medicine, Bronx, NY 10461, USA
[5]Department of Biochemistry and Molecular Biomedicine, Faculty of Biology and Institute of Biomedicine of the University of Barcelona, University of Barcelona, Barcelona 08028, Spain
[6]Instituto de Investigación Sanitaria del Hospital Clínico San Carlos (IdISSC), Madrid 28040, Spain
[7]Institut National de la Santé et de la Recherche Médicale (INSERM) and University of Bordeaux, NeuroCentre Magendie, Physiopathologie de la Plasticité Neuronale, U1215, Bordeaux 33077, France
[8]Department of Anatomy, Histology and Neuroscience, School of Medicine, Autónoma University, Madrid 28029, Spain
[9]Department of Psychiatry and Behavioral Sciences, Albert Einstein College of Medicine, Bronx, NY 10461, USA

*To whom correspondence should be addressed:
Manuel Guzmán, PhD
Department of Biochemistry and Molecular Biology
Instituto Universitario de Investigación Neuroquímica (IUIN)
Complutense University, Madrid 28040, Spain
Email: mguzman@quim.ucm.es

This Supplementary Information file includes:

- **Supplementary Tables 1 and 2**
- **Supplementary Figures 1, 2, 3, 4, 5, 6 and 7**
- **Supplementary Methods**

Protein Name	Score	Unique peptides	Coverage
Serum albumin	135.42	9	22.41
Cannabinoid receptor 1	71.16	2	9.49
LanC-like protein 2	44.24	7	17.23
Heat shock cognate 71 kDa protein	41.91	3	25.42
DNA damage-binding protein 1	36.54	4	5.41
Glycodelin	21.88	1	6.18
Desmoglein-1	17.97	2	2.77
Aldehyde dehydrogenase 9 family. member A1	16.85	4	7.69
F-box only protein 3	15.70	2	11.52
Protein cereblon	14.97	3	9.01
Creatine kinase B-type	14.95	1	12.88
Lumican	12.90	3	8.5
Desmocollin-1	10.03	2	3.57
Desmoplakin	9.61	3	1.36
Immunoglobulin heavy variable 3-23	9.53	1	16.49
Filaggrin-2	8.30	2	1.74
Neuromodulin/GAP43	7.94	1	8.29
Dihydrolipoyl dehydrogenase. mitochondria	7.41	1	3.51
Complement factor B	5.34	1	7.77
Synaptic vesicle glycoprotein 2A	4.52	1	1.75
Serpin B12	4.47	1	8.74
Sodium- and chloride-dependent GABA transporter 3	4.39	1	12.88
Submaxillary gland androgen-regulated protein 3B	4.22	1	37.5
Kynurenine--oxoglutarate transaminase 3	4.14	1	2.79
Protein farnesyltransferase/geranylgeranyltransferase type-1 subunit α	4.10	1	22.41
Cartilage acidic protein 1	4.00	1	7.25
Glial fibrillary acidic protein	3.93	1	8.47
Corneodesmosin	3.93	1	16.36
Secretory phospholipase A2 receptor	3.78	1	1.08
Protein S100-A8	3.61	1	11.83
Glyceraldehyde-3-phosphate dehydrogenase	3.61	1	6.01
Kelch repeat and BTB domain-containing protein 11	3.58	1	2.4
Lactadherin	3.54	1	3.4
Guanine nucleotide-binding protein G(i) subunit alpha-1	3.46	1	3.26
Plasma membrane calcium-transporting ATPase 1	3.44	1	8.66
ATP-dependent 6-phosphofructokinase. muscle type	3.28	1	2.28
Dermcidin	3.25	1	10
Mitogen-activated protein kinase 1	3.17	1	6
EEF1A1 protein	3.17	1	16.98
Tubulin alpha-8 chain	3.08	1	16.22
Neutrophil defensin 1	2.97	1	30
ATP synthase subunit beta	2.96	1	10.1
Lysozyme C	2.74	1	40
Myelin proteolipid protein	2.69	1	16.42
Catalase	2.68	1	1.71
Solute carrier family 7 member 13	0.00	1	5.2
Sushi repeat-containing protein SRPX2	0.00	1	40.59
Pyridoxal kinase	0.00	1	11.58
Neurexin-2	0.00	1	2.8

Supplementary Table 1: List of potential CB_1R-interacting proteins in the proteomic-MS analyses. Hits with statistical significance are ordered by score.

Species, strain, sex	Designation	Reference (doi)	Additional information
Mus musculus C57BL/6N Male and female	*Cnr1*$^{floxed/floxed}$ (herein termed *Cnr1*$^{fl/fl}$)	Marsicano et al., 2002 (10.1038/nature00839)	Mutant mice in which the CB_1 receptor-encoding gene (*Cnr1*) has been flanked by *loxP* sequences in all body cells. They were used in this study to determine the neuroanatomical distribution of CB_1R-GAP43 complexes (Fig. 3).
Mus musculus C57BL/6N Male and female	*Cnr1*$^{fl/fl}$;*CMV*^{Cre} (herein termed *Cnr1*$^{-/-}$)	Marsicano et al., 2002 (10.1038/nature00839)	Mutant mice in which the CB_1 receptor-encoding gene (*Cnr1*) has been deleted from all body cells. They were used in this study to determine the neuroanatomical distribution of CB_1R-GAP43 complexes (Figs. 1 and 3).
Mus musculus C57BL/6N Male and female	*Cnr1*$^{fl/fl}$;*Nex1*^{Cre} (herein termed Glu-*Cnr1*$^{-/-}$)	Monory et al., 2006 (10.1016/j.neuron.2006.07.006) Soria-Gómez et al. 2014 (10.1038/nn.3647)	Conditional mutant mice in which the CB_1 receptor-encoding gene (*Cnr1*) has been selectively deleted from glutamatergic neurons of the dorsal telencephalon *sensu lato* (including neocortex, paleocortex and archicortex). They were used in this study to determine the neuroanatomical distribution of CB_1R-GAP43 complexes (Figs. 3 and S1).
Mus musculus C57BL/6N Male and female	*Cnr1*$^{fl/fl}$;*Dlx5/6*^{Cre} (herein termed GABA-*Cnr1*$^{-/-}$)	Monory et al., 2006 (10.1016/j.neuron.2006.07.006)	Conditional mutant mice in which the CB_1 receptor-encoding gene (*Cnr1*) has been selectively deleted from GABAergic neurons of the forebrain. They were used in this study to determine the neuroanatomical distribution of CB_1R-GAP43 complexes (Figs 3. and S1).
Mus musculus C57BL/6N Male and female	*Cnr1*$^{stop/stop}$ (herein termed Stop-*Cnr1*)	Ruele et al., 2013 (10.1523/JNEUROSCI.4171-12.2013)	Mutant mice in which the CB_1 receptor-encoding gene (*Cnr1*) has been inactivated in all body cells owing to the insertion of a *loxP*-flanked stop cassette in the 5′-UTR upstream of the *Cnr1* gene translation start codon. They were used in this study to determine the neuroanatomical distribution of CB_1R-GAP43 complexes (Fig. 3).
Mus musculus C57BL/6N Male and female	*Cnr1*$^{stop/stop}$;*EIIa*^{Cre} (herein termed *Cnr1*-RS)	Ruele et al., 2013 (10.1523/JNEUROSCI.4171-12.2013)	Mutant mice in which the expression of the CB_1 receptor-encoding gene (*Cnr1*) has been rescued from a *Cnr1*-null background in all body cells. They were used in this study to determine the neuroanatomical distribution of CB_1R-GAP43 complexes (Fig. 3).
Mus musculus C57BL/6N Male and female	*Cnr1*$^{stop/stop}$;*Nex1*^{Cre} (herein termed Glu-*Cnr1*-RS)	Ruele et al., 2013 (10.1523/JNEUROSCI.4171-12.2013)	Mutant mice in which the expression of the CB_1 receptor-encoding gene (*Cnr1*) has been rescued from a *Cnr1*-null background selectively in glutamatergic neurons of the dorsal telencephalon *sensu lato* (including neocortex, paleocortex and archicortex). They were used in this study to determine the neuroanatomical distribution of CB_1R-GAP43 complexes (Fig. 3).

Mus musculus C57BL/6N Male and female	*Cnr1*$^{stop/stop}$;*Dlx5/6*^{Cre} (herein termed GABA-*Cnr1*-RS)	Remers et al., 2017 (10.1007/s00429-017-1411-5)	Mutant mice in which the expression of the CB_1 receptor-encoding gene (*Cnr1*) has been rescued from a *Cnr1*-null background selectively in GABAergic neurons of the forebrain. They were used in this study to determine the neuroanatomical distribution of CB_1R-GAP43 complexes (Fig. 3).
Mus musculus C57BL/6N Male and female	*Gap43*$^{floxed/floxed}$ (herein termed *Gap43*$^{fl/fl}$)	This study [generated by crossing the B6Dnk;B6Brd;B6N-Tyrc-Brd *Gap43*$^{tm1a(EUCOMM)Wtsi/Wtsi Biat}$ mouse line (EMMA Mouse Repository, MGI ID 5700649) with the ACTB:FLPe line (The Jackson Laboratory, strain #005703)]	Mutant mice in which the GAP43-encoding gene (*Gap43*) has been flanked by *loxP* sequences in all body cells. They were used in this study to determine (*i*) the neuro-anatomical distribution of CB_1R-GAP43 complexes (Figs. S2 and S3), (*ii*) the role of GAP43 in hippocampal synaptic plasticity (Fig. 5), and (*iii*) the role of GAP43 in behavioral and EEG parameters (Figs. 6, S3 and S5).
Mus musculus C57BL/6N Male and female	*Gap43*$^{fl/fl}$;*Nex1*^{Cre} (herein termed Glu-*Gap43*$^{-/-}$)	This study	Conditional mutant mice in which the GAP43-encoding gene (*Gap43*) has been selectively deleted from glutamatergic neurons of the dorsal telencephalon *sensu lato* (including neocortex, paleocortex and archicortex). They were used in this study to determine (*i*) the neuro-anatomical distribution of CB_1R-GAP43 complexes (Figs. S2 and S3), and (*ii*) the role of GAP43 in behavioral and EEG parameters (Figs. 6 and S3).
Mus musculus C57BL/6N Male and female	*Gap43*$^{fl/fl}$;*Dlx5/6*^{Cre} (herein termed GABA-*Gap43*$^{-/-}$)	This study	Conditional mutant mice in which the GAP43 receptor-encoding gene (*Gap43*) has been selectively deleted from GABAergic neurons of the forebrain). They were used in this study to determine (*i*) the neuro-anatomical distribution of CB_1R-GAP43 complexes (Figs S2 and S3), and (*ii*) the role of GAP43 in behavioral and EEG parameters (Figs. 6, S3 and S5).

Supplementary Table 2: List of the mutant-mouse lines used in this study. The lines were generated by the *Cre-loxP* technology using non-selective promoters (either the human cytomegalovirus promoter or the adenoviral *EIIa* promoter) or neuron population-selective promoters (either the glutamatergic-neuron, dorsal-telencephalon promoter *Nex1* or the GABAergic-neuron, forebrain promoter *Dlx5/6*).

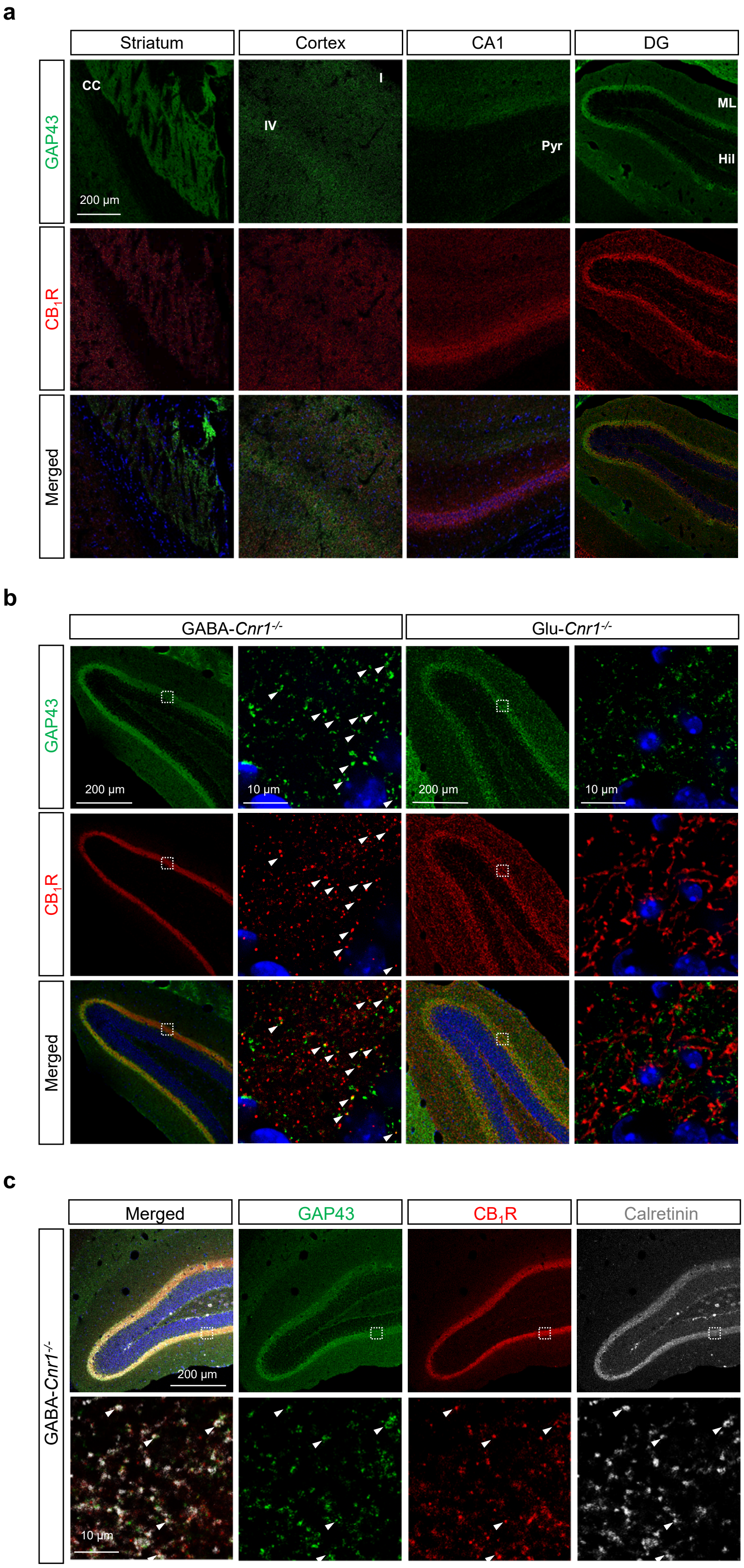

Supplementary Fig. 1: CB_1R and GAP43 colocalize in MC axon terminals of the DG. a GAP43 and CB_1R immunoreactivity in sections from striatum, cortex, and hippocampal CA1 area and DG of 3-month-old WT mice. Nuclei are stained with DAPI (blue). Representative images are shown. The experiment was repeated independently 3 times with similar results. CC: corpus callosum; I-IV: cortical layers I-IV; Pyr: pyramidal layer; ML: molecular layer; Hil: hilus. **b** GAP43 and CB_1R immunoreactivity in the DG of GABA-*Cnr1*$^{-/-}$ (*left*) and Glu-*Cnr1*$^{-/-}$ (*right*) mice. Nuclei are stained with DAPI (blue). The dotted line depicts the high-magnification inset of the IML shown in the corresponding right-hand columns. Arrowheads point to some of the colocalizing boutons in GABA-*Cnr1*$^{-/-}$ mice, which were absent in Glu-*Cnr1*$^{-/-}$ animals. Representative images are shown. The experiment was repeated independently 4 times with similar results. **c** Triple labelling of GAP43, CB_1R and calretinin immunoreactivity in the DG of GABA-*Cnr1*$^{-/-}$ mice. Nuclei are stained with DAPI (blue). The dotted line depicts the high-magnification inset of the IML shown below. Arrowheads point to some of the colocalizing boutons. Representative images are shown. The experiment was repeated independently 3 times with similar results.

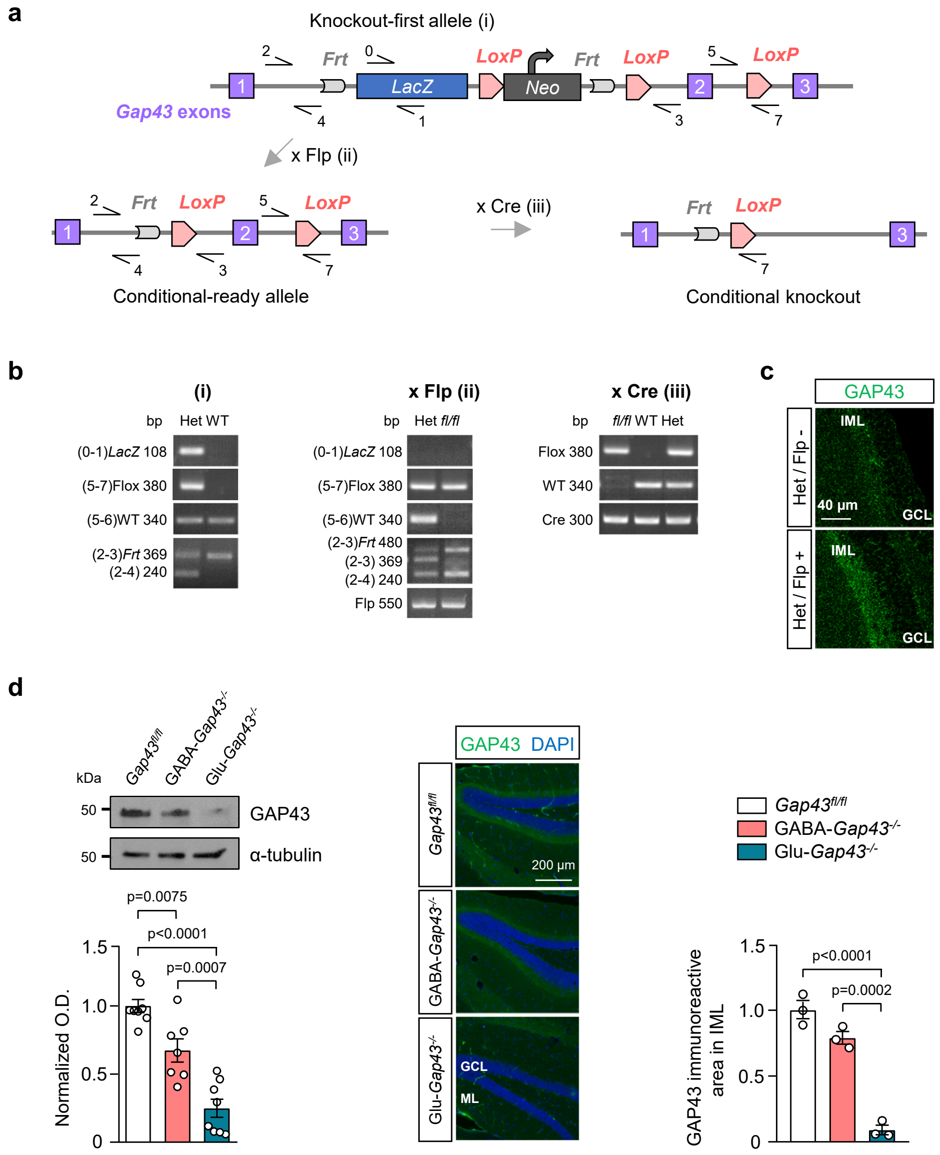

Supplementary Fig. 2: Generation of conditional *Gap43*$^{-/-}$ mouse lines. a Gene targeting strategy used to generate conditional *Gap43*$^{-/-}$ mice. Gap43 reporter knockout-first allele heterozygous (step i) required a first cross with mice carrying the *Actb-Flp* transgene, which encodes a constitutively-expressed Flp recombinase to remove the *LacZ* and *Neo* cassettes, thus allowing the expression of floxed exon 2 in the *Gap43* gene (step ii) to achieve a conditional-ready allele. The resulting heterozygotes were positive for Flp recombinase and the *Frt*-flanked sequence deletion, as tested by tail-snip PCR with the indicated primers (black arrows). These animals were finally backcrossed to obtain the homozygous floxed line (hereafter *Gap43*$^{fl/fl}$ mice). Subsequent crossing with Cre-expressing mice (step iii) yielded the conditional knockout mice in the specific locations of Cre recombinase expression. **b** Representative PCR genotyping of (step i) heterozygous or WT allele before *Flp*-mouse crossing (the heterozygous allele would harbor *LacZ*, *Frt* and *LoxP* sequences), (step ii) heterozygous or homozygous alleles after *Flp*-mouse crossing, and (step iii) *Gap43*$^{fl/fl}$ and *Dlx5/6*-Cre or *Nex1*-Cre alleles. This procedure was performed for every mouse in the colony. **c** Re-expression of GAP43 in the IML of knockout-first allele heterozygous mice crossed with *Flp*-mice. GCL: granule cell layer. The experiment was repeated independently 3 times with similar results. **d** *Left,* Representative Western blot of GAP43 protein in hippocampal extracts from 3-month-old *Gap43*$^{fl/fl}$, GABA-*Gap43*$^{-/-}$ and *Glu-Gap43*$^{-/-}$ mice, and quantification of normalized optical density (O.D.) values of GAP43 relative to those of the loading control, are shown (means ± SEM; *Gap43*$^{fl/fl}$ n = 8 mice, *Glu-Gap43*$^{-/-}$ n = 8 mice, GABA-*Gap43*$^{-/-}$ n = 7 mice; one-way ANOVA with Tukey's multiple comparisons test). *Right,* Reduced GAP43 immunoreactivity in the IML of Glu-*Gap43*$^{-/-}$ mice compared to *Gap43*$^{fl/fl}$ or GABA-*Gap43*$^{-/-}$ mice (means ± SEM; n = 3 mice per group; one-way ANOVA with Tukey's multiple comparisons). GCL: granule cell layer; ML: molecular layer. Source data are provided as a Source Data file.

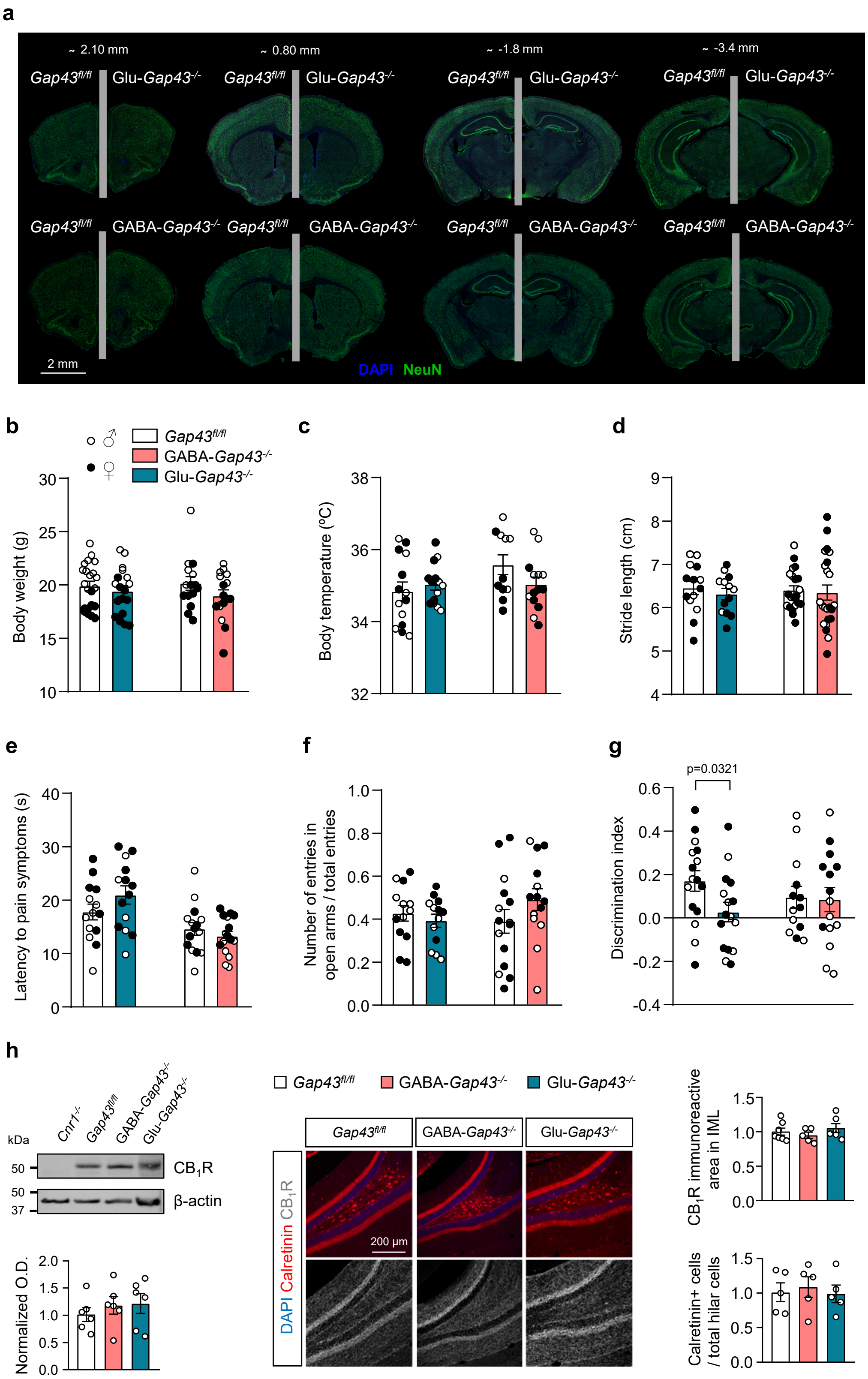

Supplementary Fig. 3: General characterization of conditional *Gap43*$^{-/-}$ mouse lines. a DAPI and NeuN co-staining of coronal slices showing a normal gross morphology of the brain for both genotypes. Representative stitched images and approximate coordinates to bregma are shown. The experiment was repeated independently 3 times with similar results. **b-g** Glu-*Gap43*$^{-/-}$ and GABA-*Gap43*$^{-/-}$ mice vs their *Gap43*$^{fl/fl}$ littermates at *ca.* 3 months of age were analyzed. No significant differences (by two-tailed unpaired Student's *t*-test) were found in body weight (**b**, means ± SEM; Glu-*Gap43*$^{fl/fl}$ n = 22 mice, Glu-*Gap43*$^{-/-}$ n = 18 mice, GABA-Gap43$^{fl/fl}$ n = 15 mice, GABA-*Gap43*$^{-/-}$ n = 16 mice), body temperature (**c**, means ± SEM; Glu-*Gap43*$^{fl/fl}$ n = 14 mice, Glu-*Gap43*$^{-/-}$ n = 14 mice, GABA-*Gap43*$^{-/-}$ n = 14 mice, GABA-*Gap43*$^{fl/fl}$ n = 11 mice), gait pattern (**d**, means ± SEM; Glu-*Gap43*$^{fl/fl}$ n = 14 mice, Glu-*Gap43*$^{-/-}$ n = 13 mice, GABA-Gap43$^{fl/fl}$ n = 21 mice, GABA-*Gap43*$^{-/-}$ n = 23 mice), nociceptive response (**e**, means ± SEM; Glu-*Gap43*$^{fl/fl}$ n = 14 mice, Glu-*Gap43*$^{-/-}$ n = 14 mice, GABA-Gap43$^{fl/fl}$ n = 15 mice, GABA-*Gap43*$^{-/-}$ n = 16 mice), or anxious behavior (**f**, means ± SEM; Glu-*Gap43*$^{fl/fl}$ n = 13 mice, Glu-*Gap43*$^{-/-}$ n = 13 mice, GABA-Gap43$^{fl/fl}$ n = 15 mice, GABA-*Gap43*$^{-/-}$ n = 14 mice). **g** Novel object recognition test showing an impairment of memory in Glu-*Gap43*$^{-/-}$ mice (means ± SEM; Glu-*Gap43*$^{fl/fl}$ n = 16 mice, Glu-*Gap43*$^{-/-}$ n = 16 mice, GABA-Gap43$^{fl/fl}$ n = 13 mice, GABA-*Gap43*$^{-/-}$ n = 15 mice; two-tailed unpaired Student's *t*-test). **h** CB_1R levels were unaltered in the whole hippocampus (*left,* means ± SEM; n = 6 mice per group; n.s. by one-way ANOVA with Tukey's multiple comparisons test) or specifically in the IML (*middle* and *right,* means ± SEM; *Gap43*$^{fl/fl}$ n = 7 mice, GABA-*Gap43*$^{-/-}$ n = 5 mice, Glu-*Gap43*$^{-/-}$ n = 6 mice; n.s. by one-way ANOVA with Tukey's multiple comparisons test). No significant differences were found in the number of calretinin-expressing MCs in the hilus from *Gap43*$^{fl/fl}$, GABA-*Gap43*$^{-/-}$ or Glu-*Gap43*$^{-/-}$ mice (*middle* and *right,* means ± SEM; n = 5 mice per group; n.s. by one-way ANOVA with Tukey's multiple comparisons test). Source data are provided as a Source Data file.

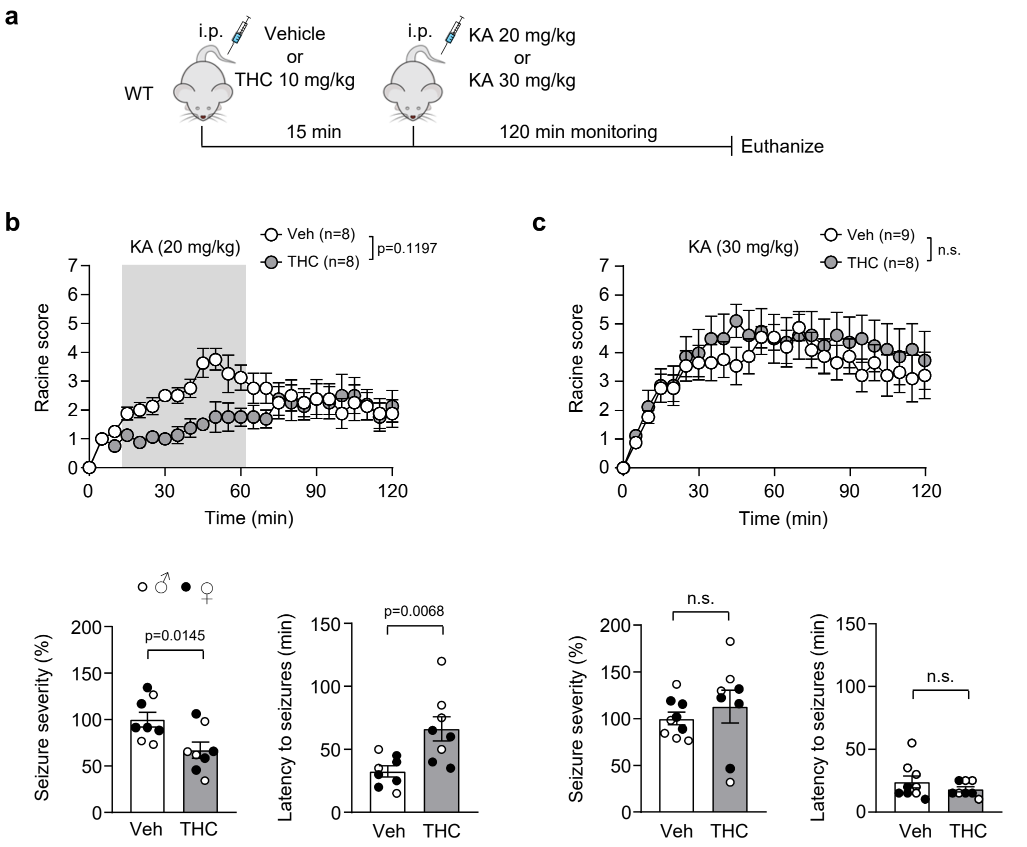

Supplementary Fig. 4: Controls of drug dosing of the excitotoxic-seizures experiments. **a** Timeline of the experiments. Vehicle or THC (10 mg/kg, i.p.; 1 injection) was administered to 3-month-old WT mice. Kainic acid (KA; 20 or 30 mg/kg, i.p.; 1 injection) was administered 15 min later, and behavioral score was monitored for 120 min. **b** *Top*, Behavioral scoring of seizures at KA 20 mg/kg using a modified Racine scale (means ± SEM; number of animals in parentheses; the shaded area indicates all the time points at which $p < 0.05$ by two-way ANOVA with Sidak's multiple comparisons test). *Down*, Integrated seizure severity, expressed as normalized percentage from Vehicle group, and latency to seizures (means ± SEM; n = 8 mice per group; two-tailed unpaired Student´s *t*-test). **c** *Top*, Behavioral scoring of seizures at KA 30 mg/kg using a modified Racine scale (means ± SEM; number of animals in parentheses; n.s. by two-way ANOVA with Sidak's multiple comparisons). *Bottom*, Integrated seizure severity, expressed as normalized percentage from Vehicle group, and latency to seizures (means ± SEM; Vehicle n = 9 mice, THC n = 8 mice; n.s. by two-tailed unpaired Student´s *t*-test). Source data are provided as a Source Data file.

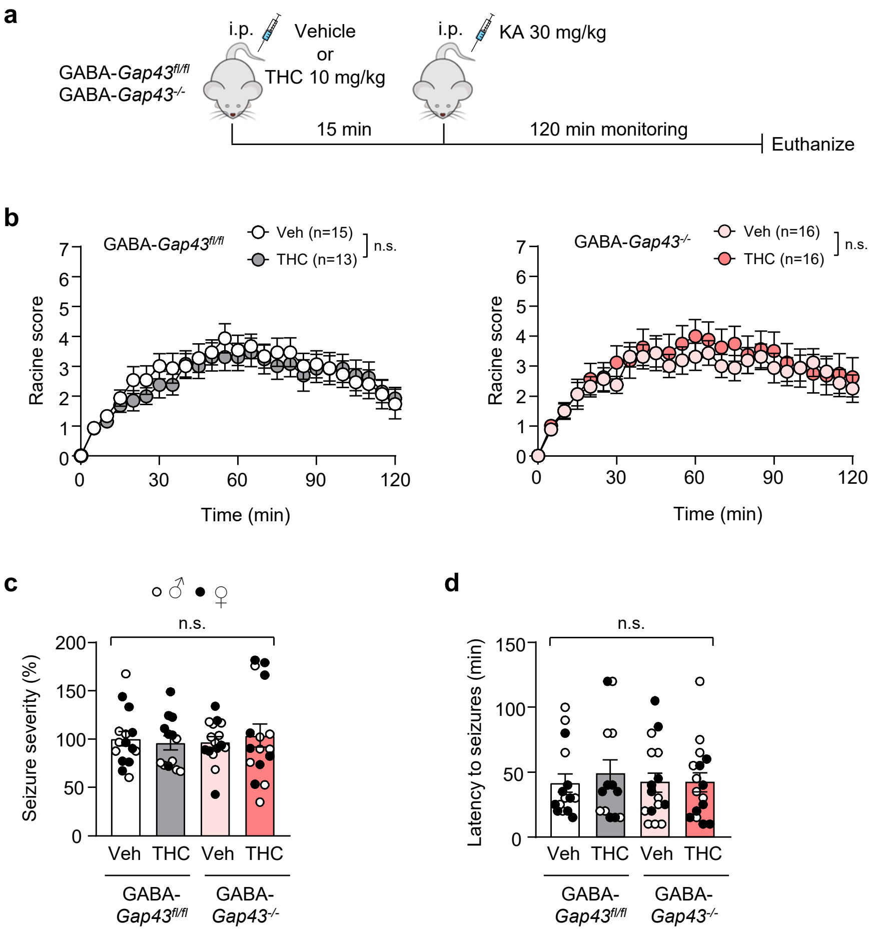

Supplementary Fig. 5: Lack of anti-convulsant response to THC in GABA-*Gap43*$^{-/-}$ mice. **a** Timeline of the experiments. Vehicle or THC (10 mg/kg, i.p.; 1 injection) was administered to 3-month-old GABA-*Gap43*$^{-/-}$ mice and their corresponding *Gap43*$^{fl/fl}$ littermates. Kainic acid (KA; 30 mg/kg, i.p.; 1 injection) was administered 15 min later, and behavioral score was monitored for 120 min. **b** Behavioral scoring of seizures using a modified Racine scale (means ± SEM; number of animals in parentheses; n.s. by two-way ANOVA with Sidak's multiple comparisons test). **c** Integrated seizure severity, expressed as normalized percentage from *Gap43*$^{fl/fl}$ / Vehicle group (means ± SEM; *Gap43*$^{fl/fl}$ / Vehicle n = 15 mice, *Gap43*$^{fl/fl}$ / THC n = 13 mice, GABA-*Gap43*$^{-/-}$ / Vehicle n = 16 mice, GABA-*Gap43*$^{-/-}$ / THC n = 16 mice; n.s. by two-way ANOVA with Tukey's multiple comparisons test). **d** Latency to seizures (means ± SEM; *Gap43*$^{fl/fl}$ / Vehicle n = 15 mice, *Gap43*$^{fl/fl}$ / THC n = 13 mice, GABA-*Gap43*$^{-/-}$ / Vehicle n = 16 mice, GABA-*Gap43*$^{-/-}$ / THC n = 16 mice; n.s. by two-way ANOVA with Tukey's multiple comparisons test). Source data are provided as a Source Data file.

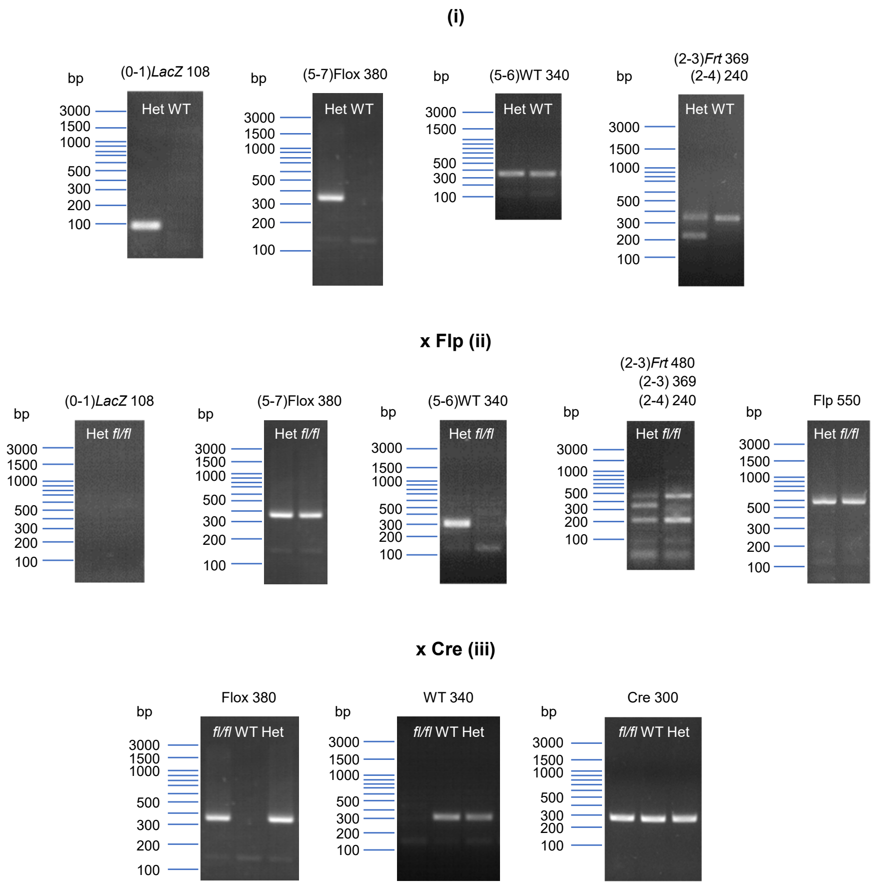

Supplementary Fig. 6: Uncropped agarose gels from Supplementary Fig. 2b.

Supplementary Fig. 2d

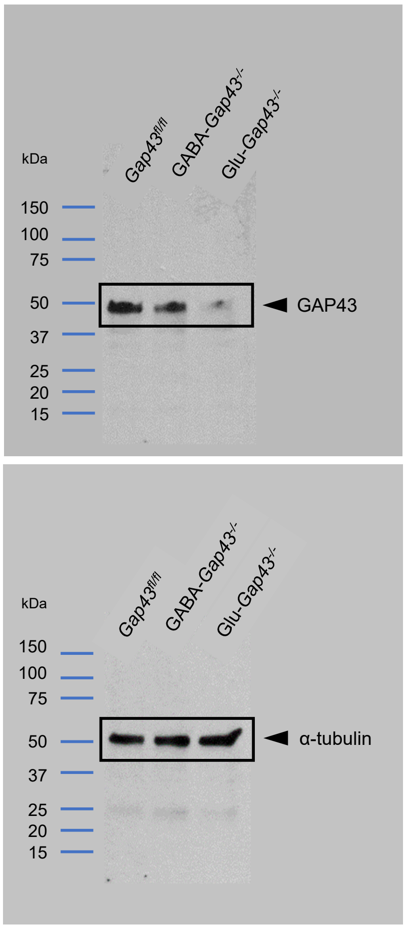

Supplementary Fig. 3h

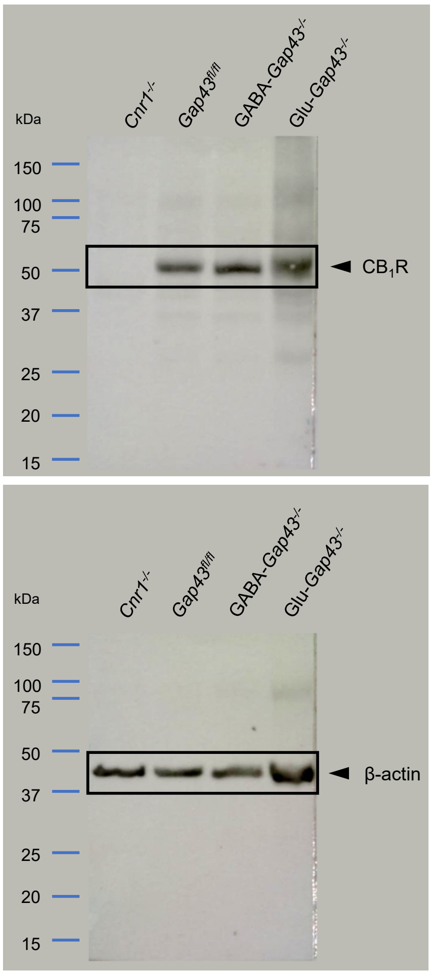

Supplementary Fig. 7: Uncropped Western blots from Supplementary Figs. 2d and 3h.

SUPPLEMENTARY METHODS

Gene constructs

The recombinant expression of hCB_1R *C*-terminal domain (CTD) for liquid chromatography-coupled mass spectrometry was obtained upon cloning this fragment into the expression vector pKLSLt (kindly given by Dr. José M. Mancheño, IQFR, CSIC, Madrid, Spain), containing an in-frame lectin cDNA from *Laetiporus sulphurous*. Expression of hGAP43 and hCB_1R-CTD for fluorescence polarization assays was performed by cloning the gene into the pBH4 plasmid with an *N*-terminal 6xHis tag previously used in our laboratory[1]. The pEGFP-C2 plasmid was purchased from ClonTech (Mountain View, CA, USA). We generated pEGFP-hGAP43, pEGFP-hGAP43(S41D) and pEGFP-hGAP43(S41A) by using standard cloning procedures. The hGAP43-encoding cDNA was obtained from DNAsu (Clone ID HsCD00042260). The pcDNA3.1(+) plasmid was purchased from Invitrogen (Waltham, MA, USA). pcDNA3.1-HA-CB_1R, pcDNA3.1-CB_1R-myc and CB_1R-Rluc constructs had been generated previously in our laboratory. pcDNA-hGAP43, pcDNA-hGAP43(S41D), pcDNA-hGAP43(S41A), pRLuc-CB_1R and AAV expression vectors were built by conventional PCR and restriction cloning using pcDNA3.1, pRLuc-N1 or pAM-CBA-CFP as plasmid backbones, respectively. All plasmids were validated by Sanger sequencing before use.

Protein expression and purification

pBH4 plasmids encoding 6xHis-tagged hGAP43 or CB_1R-CTD (amino acids 400-472) were used to transform competent BL21 DE3 *Escherichia coli* by a heat shock procedure. For recombinant protein expression, bacteria were subsequently inoculated in 2xYT media (1.6 % w/v tryptone, 1 % w/v yeast extract, and 5 g/L NaCl, pH 7.0) at 37 °C and constant agitation. Protein expression was induced by addition of 0.5 mM isopropyl 1-thio-β-D-galactopyranoside (Panreac Química SAU, Barcelona, Spain). For protein purification, bacteria were pelleted by centrifugation at 5000*g* for 15 min at room temperature and resuspended in ice-cold lysis buffer (100 mM Tris-HCl, 100 mM NaCl, 10 mM imidazole, pH 7.0) with continuous shaking in the presence of protease inhibitors (1 mg/mL aprotinin, 1 mg/ mL leupeptin, 200 mM PMSF), 0.2 g/L lysozyme, and 5 mM β-mercaptoethanol, followed by four cycles of sonication on ice. The lysate was subsequently clarified by centrifugation at 12,000*g* for 30 min at 4° C and filtered through porous paper. Recombinant 6xHis-tagged proteins were sequentially purified on a nickel-nitrilotriacetic acid affinity column. After extensive washing (50 mM Tris-HCl, 100 mM NaCl, 25 mM imidazole, pH 7.0), proteins were eluted with elution buffer (50 mM Tris-

HCl, 100 mM NaCl, 250 mM imidazole, pH 7.0; supplemented with the aforementioned protease inhibitors). Protein purity was confirmed by SDS-PAGE and Coomassie Brilliant Blue or Silver staining. Pure protein solutions were concentrated by centrifugation in Centricon tubes (Millipore).

Fluorescence polarization

6xHis-tagged CB_1R-CTD was labeled with 5-(iodoacetamido)fluorescein (5-IAF) by standard procedures. Briefly, the dye was dissolved in DMSO, and the labeling reaction was performed in sodium bicarbonate buffer, pH 9.0, with a 3-fold molar excess of dye for 1 h at 25 °C, protected from light. Subsequently, a 1.00-Da cutoff dialysis membrane was used to eliminate the nonreacted 5-IAF. After extensive dialysis, the concentration of the labeled peptide was calculated by using the value of 68,000 cm^{-1} M^{-1} as the molar extinction coefficient of the dye at pH 8.0, and a wavelength of 494 nm. Saturation binding experiments were performed essentially as described previously[1,2], with a constant concentration of 100 nM 5-IAF-CB_1R-CTD and increasing amounts of GAP43 (0-300 μM), and 3 internal replicates per point within each experiment. The fluorescence polarization values obtained were fitted to the equation $(FP - FP_0) = (FP_{max} - FP_0)[GAP43]/(K_d + [GAP43])$, where FP is the measured fluorescence polarization, FP_{max} the maximal fluorescence polarization value, FP_0 the fluorescence polarization in the absence of added GAP43, and K_d the dissociation constant, as determined with GraphPad Prism version 8.0.1 (GraphPad Software, San Diego, CA, USA).

Cell culture and transfection

The HEK-293T cell line was purchased from the American Type Culture Collection (Manassas, VA, USA). Cells were grown in DMEM supplemented with 10% v/v FBS, 1 mM Na-pyruvate, 2 mM L-glutamine, 1% streptomycin/penicillin, and essential medium non-essential amino acids solution (diluted 1/100) (all from Invitrogen). Cells were maintained at 37 °C in an atmosphere with 5% CO_2. Cells growing in 100 mm-diameter dishes were transfected with the corresponding protein-encoding cDNA by the polyethylenimine method (Sigma-Aldrich, Steinheim, Germany) in a 4:1 mass ratio to DNA. Double transfections were performed with equal amounts of the two plasmids. In all cases, cells were washed twice with PBS, detached, and harvested for further procedures, 48 h after transfection.

Western blotting and co-immunoprecipitation

Samples for Western blotting were prepared on ice-cold lysis buffer (50 mM Tris-HCl, 0.1% Triton X-100, 1 mM EDTA, 1 mM EGTA, 50 mM NaF, 10 mM Na-β-glycerophosphate, 5 mM Na-pyrophosphate, and 1 mM Na-orthovanadate, pH 7.5; or 25 mM Tris-HCl, 140 mM NaCl, 2 mM EDTA, 5 mM $MgCl_2$, and 0.5% w/v n-dodecyl β-D-maltoside, pH 7.4, for CB_1R detection) supplemented with a protease-inhibitor cocktail (Roche, Basel, Switzerland), 0.1 mM PMSF, 0.1% β-mercaptoethanol, and 1 μM microcystin. Cell lysates were clarified by centrifugation at 12,000*g* for 15 min (4 °C), and total protein concentration was determined by the Bradford assay. Then, 20-50 μg of total protein aliquots was mixed with 5X Laemmli sample buffer and boiled at 95 °C for 5 min (except for CB_1R detection, in which samples were heated at 55 ºC for 10 min)[3]. Equal amounts of protein samples were electrophoretically resolved on 12 % SDS–PAGE and transferred to PVDF membranes (Whatman Schleicher Schuell, Keene, NH, USA). After incubation for 1 h in 5 % w/v BSA in TBS-Tween-20 (0.1 %), membranes were blotted overnight at 4 °C with the following primary antibodies and dilutions: rabbit anti-HA (1:1000, Cell Signaling Technology, #3724), guinea pig anti-CB_1R (1:1000, Frontier Institute, #CB1-GP-Af530), mouse anti-α-tubulin (clone DM1A; 1:10000, Sigma-Aldrich, #T9026), mouse anti-β-actin (clone AC-15; 1:5000, Sigma-Aldrich, #A5441), and mouse anti-*pan*-GAP-43 antibody (clone 7B10; 1:1000, Santa Cruz Biotechnology, #sc-33705), which recognizes all post-translationally modified forms of GAP43[4,5]. All antibodies were prepared in TBS-Tween-20 (0.1 %) with 5% w/v BSA. PVDF membranes were then rinsed 3 times with TBS-Tween-20 (0.1%), and HRP-linked secondary antibodies, selected according to the species of origin of the primary antibodies (mouse IgG HRP-linked antibody, 1:5000, Sigma-Aldrich, #NA-931-1; rabbit IgG HRP-linked antibody, 1:5000, Sigma-Aldrich, #NA-934V; guinea pig IgG HRP-linked antibody, 1:2000, Thermo Scientific, #A18769), were added for 1 h TBS-Tween-20 (0.1 %) at room temperature. After washing 3 times for 10 min with TBS-Tween-20 (0.1 %), membranes were developed with an enhanced chemiluminescence kit (Bio-Rad). Densitometric analysis of the relative expression of the protein of interest *vs.* the corresponding loading control was performed with ImageJ open-source software version 1.52n (NIH, Bethesda, MD). Gel exposure was adjusted to allow an appropriate densitometric analysis. Western blot images were cropped for clarity. Electrophoretic migration of molecular weight markers is depicted on the left-hand side of each blot.

For co-immunoprecipitation, primary mouse-neuron and mouse-hippocampus samples were solubilized in lysis buffer (10 mM Tris-HCl, pH 8.0, 150 mM NaCl, and 10% v/v glycerol) containing 0.5% v/v NP-40 plus a cocktail of protease and phosphatase inhibitors (protease inhibitor cocktail: 0.5 mM PMSF, 1 mM NaF, 1 mM Na-molybdate,

and 0.5 mM Na-orthovanadate). Cell lysates were clarified by centrifugation at 12,000*g* for 15 min (4 °C), and total protein was quantified with the Bradford assay. Aliquots of 20-50 µg total protein were collected to check for transfection levels (cell extract). To discard non-specific contaminants in each case 1 mg of total protein was incubated with 20 µL of Protein G-Sepharose beads (GE Healthcare Bio-Sciences, MA, USA, #17-0618-01) previously equilibrated in PBS, for 45 min in a cold room. Beads were then discarded by centrifugation (700*g*, 4 °C, 3 min), and the resulting supernatant was incubated overnight with 2 µg/sample of either mouse anti-*pan*-GAP-43 antibody (1:1000, Santa Cruz Biotechnology #sc-33705) or mouse IgG isotype control (1:1000, Invitrogen, #10400C) in a rotating-wheel at 4 °C. Finally, antibody-protein complexes were recovered by incubation with 25 µL of Protein G-Sepharose beads for 2 h followed by centrifugation with 3 in-between washing steps with lysis buffer. Samples were eluted in 30 µL of 2X Laemmli sample buffer without β-mercaptoethanol and heated for 10 min at 55 °C. Approximately a third part of the elution was further analyzed by Western blotting as described above. For HEK-293T cells, the procedure was essentially equal, but we used to 1 µg/sample of agarose covalently-coupled mouse anti-HA antibody (Thermo Scientific, #26181) and eluted samples were boiled at 95 °C for 5 min and analyzed by Western blotting.

Synaptosomal preparations

Hippocampal synaptosomes were isolated from 3-month-old *Cnr1*$^{-/-}$ mice and WT control littermates and purified on discontinuous Percoll gradients (Amersham Biosciences) as described previously[6]. Then, 1 mg of the final synaptosomal suspension was centrifuged, and the pellets containing the synaptosomes were resuspended (0.67 mg/mL, 0.32M sucrose, pH 7.4) and seeded on poly L-lysine-coated coverslips for 1 h (37 ºC), and then fixed for 5 min in 4 % paraformaldehyde (PFA) in 0.1 M phosphate buffer (pH 7.4, room temperature) for conducting subsequent immunofluorescence or PLA procedures (see below). For the latter, each experimental value was calculated as the mean of the PLA-positive immunofluorescence signal of each synaptosome in the counted fields (a minimum of 3 per sample) divided by the total number of synaptosomes in those fields (as detected by bright field microscopy). A total of 3 *Cnr1*$^{-/-}$ and 3 WT independent synaptosomal preparations were used, each preparation obtained by pooling the hippocampi of 5-6 mice. Then, the mean of the 3 WT independent preparations was set at 100%.

Immunofluorescence

Mice were perfused transcardially with PBS followed by PFA solution. Brains were dissected and post-fixed overnight in the same solution, cryoprotected with sucrose and mounted on standard cryomold with OCT compound. Serial coronal o sagittal cryostat sections (30 µm-thick) through the whole brain were collected in PBS as free-floating sections and stored at -20 °C. Slices or coverslips were permeabilized and blocked in PBS containing 0.25% Triton X-100 and 10% or 5% goat serum (Pierce Biotechnology, Rockford, IL, USA), respectively, for 1 h at RT. Primary antibodies were diluted directly into the blocking buffer, and incubated overnight at 4 °C with the following primary antibodies and dilutions: mouse anti-*pan*-GAP43 (1:1000, Santa Cruz Biotechnology, #sc-33705), guinea pig anti-CB_1R (1:500, Frontier Institute, #CB1-GP-Af530), rabbit anti-calretinin (1:500, Swant, #7697), rabbit anti-synaptophysin-1 (1:500, Synaptic Systems, #101002), and mouse anti-NeuN (clone A60; 1:500, Millipore, #MAB377). After 3 washes with PBS for 10 min, samples were subsequently incubated for 2 h at RT with the appropriate highly cross-adsorbed AlexaFluor secondary antibodies (1:500 or 1:1000, depending on the dilution of the primary antibody; all from Invitrogen: AlexaFluor 488 goat anti-mouse IgG, #A-28175; AlexaFluor 488 goat anti-rabbit IgG, #A-11008; AlexaFluor 488 goat anti-guinea pig IgG, #A-11073; AlexaFluor 546 goat anti-mouse IgG, #A-11030; AlexaFluor 546 goat anti-rabbit IgG, # A-11035; AlexaFluor 647 goat anti-rabbit IgG, #A-21244; AlexaFluor 647 goat anti-guinea pig IgG, # A-21450), together with DAPI (Roche, Basel, Switzerland) to visualize nuclei. After washing 3 times in PBS, sections were mounted onto microscope slides using Mowiol® mounting media. Confocal fluorescence images were acquired using LAS-X software version 3.4.2.18368 with an SP8 confocal microscope (Leica Microsystems, Mannheim, Germany). All quantifications were obtained from a minimum of 3 fields per section and 3 sections per condition. Images were taken using apochromatic oil-immersion 20X, 40X or 63X objective, and standard (1 Airy disc) pinhole. Immunoreactive area was measured using Fiji ImageJ software, establishing a threshold to measure only specific signal that was kept constant along the different images. For colocalization assays, the resulting binary mask was used along the built-in measure function to acquire the total immunoreactive area among all the pixels inside the binary mask overlaid on top of the original image. The obtained value was then referred to the IML area present in the optic field defined as region of interest (ROI). Data were then expressed as percentage of control. Controls were included to ensure none of the secondary antibodies produced any significant signal in preparations incubated in the absence of the corresponding primary antibodies. Representative images for each condition were prepared for figure presentation by applying brightness, contrast, and other adjustments uniformly.

Proximity ligation assay (PLA)

In situ PLA for CB_1R and GAP43 was conducted in HEK-293T cells transfected with pcDNA3.1-CB_1R-myc and pEGFP-GAP43. Controls were performed by transfecting an empty GFP-vector, in the absence of CB_1R-myc and with untagged plasmids. Cells were grown on glass coverslips and fixed in 4 % PFA for 15 min. For conducting PLA in mouse hippocampal brain slices, mice were deeply anesthetized and immediately perfused transcardially with PBS followed by 4 % PFA, postfixed and cryo-sectioned as described above. Immediately before the assay, mouse brain sections were mounted on glass slides, and washed in PBS. In all cases, complexes were detected using the Duolink *in situ* PLA Detection Kit (Sigma Aldrich) following supplier's instructions. First, samples were permeabilized in PBS supplemented with 20 mM glycine and 0.05% Triton X-100 for 5 min (cell cultures) or 10 min (mounted slices) at room temperature. Slides were next incubated with Blocking Solution (one drop per cm^2) in a pre-heated humidity chamber for 1 h at 37 ºC. Primary antibodies were diluted in Antibody Diluent Reagent from the kit [mouse anti-c-myc (clone 9E10; 1:200, Sigma-Aldrich #11667149001) and rabbit anti-GFP (1:200, Abcam, #ab290) for cell cultures; mouse anti-*pan*-GAP43 (1:100, Santa Cruz Biotechnology, #sc-33705) and rabbit anti-CB_1R (1:100, Frontier Institute, #CB1-Rb-Af380) for brain sections], and incubated overnight at 4 °C. Negative controls were performed with just one primary antibody. Ligations and amplifications were performed with In Situ Detection Reagent Red (Sigma Aldrich), stained for DAPI, and mounted. Samples were analyzed with a Leica SP8 confocal microscope and processed with Fiji ImageJ software.

For cultured cells, 22 random fields along the whole preparation were used for analysis. For each field of view, a stack of two channels (one per staining) and 30 Z-stacks with a step size of 0.5 µm were acquired. For brain tissue, 3 serial sagittal sections per animal spaced 0.24 mm apart containing the DG were used, and for each image of 9 to 13 Z-stacks with a step size of 1 µm were acquired. Cells containing one or more red dots versus total GFP-positive cells were determined for quantification in experiments with cultured cells. The total number of red dots per area of IML was determined for quantification of PLA signal in tissue slices. Nuclei and red dots were counted on the maximum projections of each image stack. The blue nuclei and red dots were segmented by subtracting the background and applying a threshold to obtain the binary image. PLA dots were counted upon restricting size and circularity. Additionally, for slices, the contrast was enhanced with the Contrast Limited Adaptive Histogram Equalization (CLAHE) plug-in, and ROIs are established for quantification. Representative images for each condition were prepared for figure presentation by applying brightness, contrast, and other adjustments uniformly.

Bioluminescence resonance energy transfer (BRET)

HEK-293T cells grown in 6-well plates were transiently co-transfected with a constant amount (0.025 µg) of a cDNA encoding CB_1R fused to Rluc protein (CB_1R-Rluc) as BRET donor and increasing amounts (0.05 to 1.5 µg) of a cDNA encoding a GAP43 form fused to GFP protein [namely GAP43(WT)-GFP, GAP43(S41A)-GFP, GAP43(S41D)-GFP] as BRET acceptor. Cells were harvested, washed, resuspended in HBSS buffer with 10 mM glucose and distributed (approximately 20 µg protein per well) in 96-well microplates. Fluorescence and BRET signal was detected and analyzed as described[2]. BRET was expressed as BRET milliunits (mBU). In BRET curves, BRET was expressed as a function of the ratio between fluorescence and luminescence (GFP/Rluc). To calculate the GFP/Rluc ratio that gives the half-maximal BRET value ($BRET_{50}$), data from saturation curves were fitted by a nonlinear regression equation assuming a single phase with GraphPad Prism software version 8.0.1. Within each experiment, incubations were carried out in triplicate for every condition.

Dynamic mass redistribution (DMR) assays

The overall cell signaling signature was determined by using an EnSpire® Multimode Plate Reader (PerkinElmer, Waltham, MA, USA) with a label-free technology as described[2]. Briefly, 24 h before the assay, HEK-293T cells expressing CB_1R were seeded at a density of 10,000 cells per well in 384-well sensor microplates with 30 µL growth medium, and cultured for 24 h (37 °C, 5 % CO_2) to obtain 70 – 80 % confluent monolayers. The sensor plate was scanned, and a baseline optical signature was recorded for 10 min before adding 10 µL of the cannabinoid receptor agonist WIN-55,212-2 (Sigma-Aldrich, 100 nM final concentration) dissolved in assay buffer (HBSS with 20 mM Hepes, pH 7.15) containing 0.1% DMSO. Then, the resulting shifts of reflected light wavelength (in pm) were analyzed with EnSpire Workstation Software version 4.10 (PerkinElmer). Each DMR experiment included a vehicle condition, which routinely gave a negligible background-signal line. Data were obtained by subtracting the corresponding vehicle-datum point from each experimental value. DMR response traces were quantified by calculating the shift of the peak values (DMR_{max}, in pm) from each time-course plot using GraphPad Prism software version 8.0.1. Each representative curve shown is the mean of 3 internal replicates.

Recombinant AAV1/2 production

Chimeric AAV serotype 1/2 virions, containing a 1:1 ratio of AAV1 and AAV2 capsid proteins with AAV2 ITRs, were used. They were produced as previously described[7,8]. Briefly, HEK-293T cells were transfected with polyethyleneimine in a 4:1 mass ratio to

DNA with the AAV *cis* plasmid, the AAV1 and AAV2 helper plasmids, and pAM-CBA-CFP, pAM-CBA-GAP43(S41A)-CFP or pAM-CBA-GAP43(S41D)-CFP for each of the viral batches required. Sixty hours after transfection, cells were harvested, and the virions were purified by iodixanol (OptiPrep)-based density gradients, consisting of decreasing concentrations of OptiPrep (54 %, 40 %, 25 %, 15 %) and refrigerated (4 °C) ultracentrifugation at 160,000*g*. Virion solutions were concentrated by centrifugation in Centricon tubes (Millipore, Burlington, MA, USA). Purity analysis was performed through SDS-PAGE with 20 µL of concentrated virion preparations per lane. The genomic titers were determined using an Applied Biosystems ABI 7500 real-time PCR cycler.

Supplementary References

1. Merino-Gracia, J., Costas-Insua, C., Canales, M. Á. & Rodríguez-Crespo, I. Insights into the C-terminal peptide binding specificity of the PDZ domain of neuronal nitric-oxide synthase. *J. Biol. Chem.* **291**, 11581–11595 (2017).

2. Costas-Insua, C. *et al.* Identification of BiP as a CB_1 receptor-interacting protein that fine-tunes cannabinoid signaling in the mouse brain. *J. Neurosci.* **41**, 7924–7941 (2021).

3. Esteban, P. F. *et al.* Revisiting CB_1 cannabinoid receptor detection and the exploration of its interacting partners. *J. Neurosci. Methods* **337**, 108680 (2020).

4. He, Q., Dent, E. W. & Meiri, K. F. Modulation of actin filament behavior by GAP-43 (neuromodulin) is dependent on the phosphorylation status of serine 41, the protein kinase C Site. *J. Neurosci.* **17**, 3515–3524 (1997).

5. Meiri, K. F., Bickerstaff, L. E. & Schwob, J. E. Monoclonal antibodies show that kinase C phosphorylation of GAP-43 during axonogenesis is both spatially and temporally restricted in vivo. *J. Cell Biol.* **112**, 991–1005 (1991).

6. Martín, R. *et al.* The metabotropic glutamate receptor mGlu7 activates phospholipase C, translocates Munc-13-1 protein, and potentiates glutamate release at cerebrocortical nerve terminals. *J. Biol. Chem.* **285**, 17907–17917 (2010).

7. Monory, K. *et al.* The endocannabinoid system controls key epileptogenic circuits in the hippocampus. *Neuron* **51**, 455–466 (2006).

8. Ruiz-Calvo, A. *et al.* Pathway-specific control of striatal neuron vulnerability by corticostriatal cannabinoid CB_1 receptors. *Cereb. Cortex* **28**, 307–322 (2018).
