# Supplementary material for: Control of a hippocampal recurrent excitatory circuit by cannabinoid receptor-interacting protein Gap43
Source: Nat Commun. 2023 Apr 21;14:2303. doi: 10.1038/s41467-023-38026-2 (PMC10121561; doi:10.1038/s41467-023-38026-2)
Supplement: Supplementary file 3 — Reporting Summary [file 41467_2023_38026_MOESM3_ESM.pdf]

nature portfolio

Corresponding author(s): Manuel Guzmán

Last updated by author(s): Mar 30, 2023

Reporting Summary

Nature Portfolio wishes to improve the reproducibility of the work that we publish. This form provides structure for consistency and transparency in reporting. For further information on Nature Portfolio policies, see our Editorial Policies and the Editorial Policy Checklist.

Statistics

For all statistical analyses, confirm that the following items are present in the figure legend, table legend, main text, or Methods section.

n/a	Confirmed	
☐	☒	The exact sample size (*n*) for each experimental group/condition, given as a discrete number and unit of measurement
☐	☒	A statement on whether measurements were taken from distinct samples or whether the same sample was measured repeatedly
☐	☒	The statistical test(s) used AND whether they are one- or two-sided<br>*Only common tests should be described solely by name; describe more complex techniques in the Methods section.*
☐	☒	A description of all covariates tested
☐	☒	A description of any assumptions or corrections, such as tests of normality and adjustment for multiple comparisons
☐	☒	A full description of the statistical parameters including central tendency (e.g. means) or other basic estimates (e.g. regression coefficient) AND variation (e.g. standard deviation) or associated estimates of uncertainty (e.g. confidence intervals)
☐	☒	For null hypothesis testing, the test statistic (e.g. *F*, *t*, *r*) with confidence intervals, effect sizes, degrees of freedom and *P* value noted<br>*Give P values as exact values whenever suitable.*
☒	☐	For Bayesian analysis, information on the choice of priors and Markov chain Monte Carlo settings
☒	☐	For hierarchical and complex designs, identification of the appropriate level for tests and full reporting of outcomes
☒	☐	Estimates of effect sizes (e.g. Cohen's *d*, Pearson's *r*), indicating how they were calculated

Our web collection on statistics for biologists contains articles on many of the points above.

Software and code

Policy information about availability of computer code

Data collection	- Peptide identification in the proteomic experiments was validated by the Percolator algorithm (built-in version of Proteome Discoverer version 1.4.1.14; Thermo Scientific).<br>- Electrophysiology data were acquired with IgorPro software version 7.01 (Wavemetrics).<br>- EEG data were collected with Spike2 software version 10 (Cambridge Electronic Design).<br>- Confocal fluorescence and proximity ligation assay images were acquired with LAS-X software version 3.4.2.18368 (Leica).<br>- Dynamic mass redistribution data were acquired with EnSpire Workstation Software version 4.10 (PerkinElmer).
Data analysis	Statistical analysis of the collected numerical data was routinely performed with GraphPad Prism version 8.0.1 (GraphPad Software). Power analysis was performed in the behavioral-seizure experiments with IBM SPSS software version 28 (IBM France, Bois-Colombes, France). In addition:<br>- Proteomic spectrum files were challenged to data bases with Proteome Discoverer software version 1.4.1.14 (Thermo Scientific) and the searching tool SEQUEST (built-in version of Proteome Discoverer version 1.4.1.14).<br>- Electrophysiology data were analyzed with IgorPro software version 7.01 (Wavemetrics).<br>- Mouse behavioral tests were analyzed with Smart3.0 Software (Panlab).<br>- EEG experiments were analyzed with Spike2 software version 10 (Cambridge Electronic Design).<br>- Western blot experiments were analyzed with ImageJ open-source software version 1.52n (NIH).<br>- Confocal fluorescence and proximity ligation assay images were analyzed with Fiji ImageJ open-source software (NIH).<br>- Shifts of reflected light wavelength in dynamic mass redistribution assays were analyzed with EnSpire Workstation Software version 4.10 (PerkinElmer).

For manuscripts utilizing custom algorithms or software that are central to the research but not yet described in published literature, software must be made available to editors and reviewers. We strongly encourage code deposition in a community repository (e.g. GitHub). See the Nature Portfolio guidelines for submitting code & software for further information.

Data

Policy information about availability of data

All manuscripts must include a data availability statement. This statement should provide the following information, where applicable:

- Accession codes, unique identifiers, or web links for publicly available datasets
- A description of any restrictions on data availability
- For clinical datasets or third party data, please ensure that the statement adheres to our policy

All data generated or analyzed during this study are included in this article, its Supplementary Information, and its Source Data file. Proteomic spectrum files were challenged to data bases and Uniprot of sheep (Ovis aries; UPID: UP000002356; accession link: https://www.uniprot.org/proteomes/UP000002356) and other mammals [Mammalian; taxonomy_id:40674; accession link: https://www.uniprot.org/proteomes?query=(taxonomy_id:40674)] using Proteome Discoverer software version 1.4.1.14 (Thermo Scientific) and the searching tool SEQUEST (built-in version of Proteome Discoverer version 1.4.1.14). Source data are provided with this paper.

Human research participants

Policy information about studies involving human research participants and Sex and Gender in Research.

Reporting on sex and gender	N/A
Population characteristics	N/A
Recruitment	N/A
Ethics oversight	N/A

Note that full information on the approval of the study protocol must also be provided in the manuscript.

Field-specific reporting

Please select the one below that is the best fit for your research. If you are not sure, read the appropriate sections before making your selection.

☒ Life sciences ☐ Behavioural & social sciences ☐ Ecological, evolutionary & environmental sciences

For a reference copy of the document with all sections, see nature.com/documents/nr-reporting-summary-flat.pdf

Life sciences study design

All studies must disclose on these points even when the disclosure is negative.

Sample size	Power analysis was performed in the behavioral-seizure experiments using IBM SPSS software version 28 (IBM France, Bois-Colombes, France). The sample size of the rest of the experiments was estimated on the basis of previous studies conducted by our laboratories using similar in vitro models (e.g., cell culture and transfection, fluorescence polarization, PLA, co-immunoprecipitation, DMR, BRET; cf. Bellocchio et al. J Neurosci 2016, 36:10611-10624; Moreno et al. Neuropsychopharmacology 2018, 43:964-977; Blasco-Benito et al. PNAS 2019, 116:3863-3872; Costas-Insua et al. J Neurosci 2021, 41:7924-7941), ex vivo models (e.g., electrophysiology/optogenetics in brain slices; cf. Jensen et al. PNAS 2021, 118, e2017590118; Nasrallah et al. PNAS 2022, 119:e2201151119) and in vivo models (e.g., immunofluorescence/PLA in synaptosomal preparations/brain sections, mouse behavior, mouse EEG; cf. Blázquez et al. Brain 2011, 134:119-136; Trueba-Sáiz et al. Transl Psychiatry 2013, 3:e330; Chiarlone et al. PNAS 2014, 111:8257-8262; Moreno et al. Neuropsychopharmacology 2018, 43:964-977; Ruiz-Calvo et al. Cereb Cortex 2018, 28:307–322; Barros-Zulaica et al. eNeuro. 2019, 6:ENEURO.0471-18.2019; Costas-Insua et al. J Neurosci 2021, 41:7924-7941).
Data exclusions	No data were excluded for the statistical analyses.
Replication	The number of biological replicates (e.g., number of mice, number of brain slices, number of cell cultures, number of cells) is provided in the corresponding figures/legends. The number of technical replicates (e.g., number of incubations per cell culture, number of microscopy sections and fields per mouse brain, number of behavioral trials per mouse) is provided in the corresponding subsections of the Methods section.
Randomization	In all experiments, biological samples (cultured cells, tissue extracts, synaptosomal preparations, brain sections, brain slices) and animals (mice) were allocated randomly into the different groups.
Blinding	All in vivo experiments (e.g., immunofluorescence/PLA in synaptosomal preparations/brain sections, mouse behavior, mouse EEG) and ex vivo experiments (e.g., electrophysiology/optogenetics in brain slices) were routinely performed and analyzed in a blinded manner for mouse genotype, pharmacological treatment and viral injection (typically, an experimenter prepared the biological samples/animals and another experimenter conducted the assays blinded to group allocation). Blinding was usually precluded for acquisition and analysis of data from in vitro experiments (e.g., cell culture and transfection, fluorescence polarization, PLA, co-immunoprecipitation, DMR, BRET) because, for

logistical reasons, it was not technically or practically feasible to do so (typically, a unique experimenter conducted the whole experimental procedure, or most of it, unblinded to group allocation). Identical use in all experiments of well-defined criteria and procedures for data acquisition and analysis reduced to a minimum the possibility that the experimenter's bias could influence results. Blinding was not applicable to proteomic experiments because they do not involve group allocation.

Reporting for specific materials, systems and methods

We require information from authors about some types of materials, experimental systems and methods used in many studies. Here, indicate whether each material, system or method listed is relevant to your study. If you are not sure if a list item applies to your research, read the appropriate section before selecting a response.

Materials & experimental systems

n/a	Involved in the study	
☐	☒	Antibodies
☐	☒	Eukaryotic cell lines
☒	☐	Palaeontology and archaeology
☐	☒	Animals and other organisms
☒	☐	Clinical data
☒	☐	Dual use research of concern

Methods

n/a	Involved in the study	
☒	☐	ChIP-seq
☒	☐	Flow cytometry
☒	☐	MRI-based neuroimaging

Antibodies

Antibodies used

The following antibodies (with their dilutions, applications, suppliers, and catalog numbers) were used in this study:
- Rabbit anti-HA (1:1000 for WB; Cell Signaling Technology, #3724)
- Guinea pig anti-CB1R (1:1000 for WB, 1:500 for IF; Frontier Institute, #CB1-GP-Af530)
- Mouse anti-alpha-tubulin (clone DM1A; 1:10000 for WB; Sigma-Aldrich, #T9026)
- Mouse anti-beta-actin (clone AC-15; 1:5000 for WB; Sigma-Aldrich, #A5441)
- Mouse anti-pan-GAP-43 antibody (clone 7B10; 1:1000 for WB, 2 ug/sample for IP, 1:1000 for IF, 1:100 for PLA; Santa Cruz Biotechnology, #sc-33705)
- Mouse IgG HRP-linked antibody (1:5000 for WB; Sigma-Aldrich, #NA-931)
- Rabbit IgG HRP-linked antibody (1:5000 for WB; Sigma-Aldrich, #NA-934)
- Guinea pig IgG HRP-linked antibody (1:2000 for WB; Thermo Scientific, #A18769)
- Mouse IgG isotype control (2 ug/sample for IP; Invitrogen, #10400C)
- Mouse anti-HA-agarose antibody (1 ug/sample for IP; Thermo Scientific, #26181)
- Rabbit anti-calretinin (1:500 for IF; Swant, #7697)
- Rabbit anti-synaptophysin-1 (1:500 for IF; Synaptic Systems, #101002)
- Mouse anti-NeuN (clone A60; 1:500 for IF; Millipore, #MAB377)
- AlexaFluor 488 goat anti-mouse IgG (1:500 or 1:1000 for IF, depending on the dilution of the primary antibody; Invitrogen, #A-28175)
- AlexaFluor 488 goat anti-rabbit IgG (1:500 or 1:1000 for IF, depending on the dilution of the primary antibody; Invitrogen, #A-11008)
- AlexaFluor 488 goat anti-guinea pig IgG (1:500 or 1:1000 for IF, depending on the dilution of the primary antibody; Invitrogen, #A-11073)
- AlexaFluor 546 goat anti-mouse IgG (1:500 or 1:1000 for IF, depending on the dilution of the primary antibody; Invitrogen, #A-11030)
- AlexaFluor 546 goat anti-rabbit IgG (1:500 or 1:1000 for IF, depending on the dilution of the primary antibody; Invitrogen, #A-11035)
- AlexaFluor 647 goat anti-rabbit IgG (1:500 or 1:1000 for IF, depending on the dilution of the primary antibody; Invitrogen, #A-21244)
- AlexaFluor 647 goat anti-guinea pig IgG (1:500 or 1:1000 for IF, depending on the dilution of the primary antibody; Invitrogen, #A-21450)
- Mouse anti-c-myc (clone 9E10; 1:200 for PLA; Sigma-Aldrich, #11667149001)
- Rabbit anti-GFP (1:200 for PLA, Abcam, #ab290)
- Rabbit anti-CB1R (1:100 for PLA, Frontier Institute, #CB1-Rb-Af380)

Validation

All antibodies were used in this study according to manufacturer's instructions and after at least preliminary testing in our laboratories. Precise information on the validation of each primary antibody for species and applications can be found in the following links:
- Rabbit anti-HA (Cell Signaling Technology, #3724)
https://www.cellsignal.com/products/primary-antibodies/ha-tag-c29f4-rabbit-mab/3724
- Guinea pig anti-CB1R (Frontier Institute, #CB1-GP-Af530)
https://nittobo-nmd.co.jp/pdf/reagents/CB1.pdf
- Mouse anti-alpha-tubulin (Sigma-Aldrich, #T9026)
https://www.sigmaaldrich.com/ES/en/product/sigma/t9026
- Mouse anti-beta-actin (1:5,000, Sigma-Aldrich, #A5441)
https://www.sigmaaldrich.com/ES/en/product/sigma/a5441
- Mouse anti-pan-GAP-43 antibody (Santa Cruz Biotechnology, #sc-33705)
https://www.scbt.com/p/gap-43-antibody-7b10
- Mouse IgG isotype control (Invitrogen, #10400C)

https://www.fishersci.es/shop/products/anti-mouse-igg-control-invitrogen/10757813
- Mouse anti-HA agarose antibody (Thermo Scientific, #26181)
https://www.thermofisher.com/order/catalog/product/26181
- Rabbit anti-calretinin (Swant #7697)
http://www.swant.com/pdfs/Rabbit_anti_calretinin_7697.pdf
- Rabbit anti-synaptophysin-1 (Synaptic Systems, #101002)
https://sysy.com/product/101002
- Mouse anti-NeuN (Millipore, #MAB377)
https://www.emdmillipore.com/US/en/product/Anti-NeuN-Antibody-clone-A60,MM_NF-MAB377
- Mouse anti-c-myc (Sigma-Aldrich, #11667149001)
https://www.sigmaaldrich.com/US/en/product/roche/roamyc
- Rabbit anti-GFP (Abcam, #ab290)
https://www.abcam.com/products/primary-antibodies/gfp-antibody-ab290.html
- Rabbit anti-CB1R (Frontier Institute, #CB1-Rb-Af380)
https://nittobo-nmd.co.jp/pdf/reagents/CB1.pdf

Eukaryotic cell lines

Policy information about cell lines and Sex and Gender in Research

Cell line source(s)	The HEK-293T cell line was purchased from the American Type Culture Collection.
Authentication	The HEK-293T cell line was not authenticated.
Mycoplasma contamination	The HEK-293T cell line was negative for mycoplasma contamination.
Commonly misidentified lines (See ICLAC register)	No misidentified cell lines were used in the study.

Animals and other research organisms

Policy information about studies involving animals; ARRIVE guidelines recommended for reporting animal research, and Sex and Gender in Research

Laboratory animals	Species: Mus musculus; strain: C57BL/6J or C57BL/6N; age: 3-14 wk. C57BL/6J wild-type mice (Charles River) were used for AAV1/2-GAP43 injections and subsequent acute hippocampal slice preparation. The rest of the experiments were conducted with the following mutant-mouse lines, all of them generated in C57BL/6N background: Cnr1floxed/floxed (Cnr1fl/fl), Cnr1floxed/floxed;CMV-Cre (Cnr1-/-), Cnr1floxed/floxed;Nex1-Cre (Glu-Cnr1-/-), Cnr1floxed/floxed;Dlx5/6-Cre (GABA-Cnr1-/-), Stop-Cnr1, Stop-Cnr1EIIa-Cre (Cnr1-RS), Stop-Cnr1Nex1-Cre (Glu-Cnr1-RS), Stop-Cnr1Dlx5/6-Cre (GABA-Cnr1-RS), Gap43floxed/floxed (Gap43fl/fl), Gap43floxed/floxed;Nex1-Cre (Glu-Gap43-/-), and Gap43floxed/floxed;Dlx5/6-Cre (GABA-Gap43-/-). The Gap43fl/fl mouse line was generated in turn by crossing the B6Dnk;B6Brd;B6N-Tyrc-Brd Gap43tm1a(EUCOMM)Wtsi/WtsiBiat line (EMMA Mouse Repository; MGI ID #5700649) with the ACTB:FLPe line (The Jackson Laboratory, strain #005703). The main characteristics of all these Cnr1 and Gap43 mutant-mouse lines, and which experiments they were used in, are shown in Supplementary Table 2.
Wild animals	The study did not involve wild animals.
Reporting on sex	Both male and female mice, at approximately 1:1 ratio within each experimental group, were used in the study. Source data were collected and analyzed as disaggregated for sex.
Field-collected samples	The study did not involve samples collected from the field.
Ethics oversight	Experimental procedures were performed in accordance with the guidelines and approval of the Animal Welfare Committees of Universidad Complutense de Madrid, Universidad Autónoma de Madrid and Comunidad de Madrid, the directives of the Spanish Government and the European Commission, as well as the guidelines of NIH and Albert Einstein College of Medicine Institutional Animal Care and Use Committee.

Note that full information on the approval of the study protocol must also be provided in the manuscript.
